# Spatiotemporal description of African swine fever virus nucleic acid and antibodies detected in pigs sampled at abattoirs in the greater Kampala metropolitan area, Uganda from May 2021 through June 2022

**DOI:** 10.1186/s40813-023-00345-7

**Published:** 2023-11-02

**Authors:** Rodney Okwasiimire, Edrine B. Kayaga, John E. Ekakoro, Dickson Ndoboli, Kate Schumann, Bonto Faburay, Aisha Nassali, Cole Hauser, Krista Ochoa, Eddie M. Wampande, Karyn A. Havas

**Affiliations:** 1 Central Diagnostic Laboratory, College of Veterinary Medicine, Animal Resources and Biosecurity, Makerere University, P.O.Box 7062, Kampala, Uganda; 2 Department of Public and Ecosystem Health, College of Veterinary Medicine, Cornell University, Ithaca, NY 14853-6401 USA; 3 Foreign Animal Disease Diagnostic Laboratory, Veterinary Services, Animal and Plant Health Inspection Services, United States Department of Agriculture, National Veterinary Services Laboratories, Greenport, NY USA

**Keywords:** African swine Fever, qPCR, Uganda, ELISA

## Abstract

**Background:**

African swine fever virus (ASFV) infections in Africa cause hemorrhagic disease in domestic pigs and is maintained by a sylvatic cycle in warthogs. It is endemic in Uganda, leading to significant economic losses. Previous studies performed in rural areas and in Kampala had differing diagnostic results. The purpose of this study was to provide a robust spatial, temporal, and diagnostic summary of pigs slaughtered in the greater Kampala metropolitan area over the course of one year. This study characterized 1208 to 1323 serum, blood, and tissue samples collected from pigs at six abattoirs in the greater Kampala metropolitan area of Uganda monthly from May 2021 through June 2022. Validated and standardized serologic and molecular diagnostics were used.

**Results:**

Only 0.15% of pigs had detectable antibodies against ASFV, suggesting low survival rates or pre-clinical diagnosis. Yet, 59.5% of pigs were positive for ASFV DNA. Blood had the lowest detection rate (15.3%) while tonsil and lymph nodes had the highest (38% and 37.5%, respectively), spleen samples (31.5%) were in between. Agreement between sample types was fair to moderate overall. A significant seasonality of ASFV infections emerged with infections found predominately in the dry seasons. Spatial assessments revealed that the greater Kampala metropolitan area abattoirs have a catchment area that overlaps with Uganda’s most pig dense regions.

**Conclusions:**

Pigs at greater Kampala metropolitan area abattoirs can be sentinels for acute disease throughout the pig dense region of Uganda, particularly in the dry seasons. The high prevalence detected suggests that pigs are sold in response to local reports of ASFV infections (panic sales). Serological surveillance is not useful, as very few pigs seroconverted in this study prior to slaughter. In contrast, tissue samples of pigs can be used to detect disease using qPCR methods.

**Supplementary Information:**

The online version contains supplementary material available at 10.1186/s40813-023-00345-7.

## Background

African swine fever virus (ASFV) is a double-stranded DNA arbovirus with a genome size ranging from 170 to 190 kb which encodes over 150 proteins, depending on the viral strain [1]. In Africa, ASFV causes a contagious hemorrhagic disease called African swine fever (ASF) in domestic pigs. The virus is often transmitted through indirect and direct contact between naïve and infected domestic pigs [2], but transmission can also occur when an *Ornithodoros moubata* feeds on them [3–5]. Further, ASFV is also maintained through a sylvatic cycle involving warthogs and *O. moubata* ticks. Domestic pig contact with infected warthogs can result in virus transmission, although it is not a significant cause of disease in the domestic pig population [5]. Infection in domestic pigs leads to food insecurity and economic losses to farmers due to the high mortality rates usually associated with outbreaks [6, 7]. ASF was first described in the early 20th century in Kenya [8] in domestic pigs. Since then, the virus has been detected in many countries in Africa and 24 genotypes have been described [9–11].

The disease is endemic in Africa, where it affects over 35 countries on the continent including Uganda [9]. Numerous studies in Uganda have characterized the genotypes detected in outbreaks as genotype IX [12–16]. A genotype X was also identified from samples stored in the United Kingdom’s Institute for Animal Health in Pirbright, England (Pirbright) that had originated in Uganda [17]. Studies at the district level have been done to further describe the disease, including an abattoir study published in 2012. The authors found a 0.2% seropositivity when using a blocking ELISA targeting the p72 antigenic protein (Ingezim PPA Compac 1.1 PPA K3) in the Mubende district with testing performed at Pirbright. This study also identified suspect cases with signs and lesions commensurate with ASFV infection [18].

Diagnostic testing for ASFV can be quite complicated and diagnostic assays used should fit the purpose of the test. In previous studies, farmers have reported disease, but few pigs tested positive for ASFV nucleic acid [19, 20]. In addition, some studies revealed a low seroprevalence [18, 19, 21], while others reported a very high seroprevalence [20]. In these studies, different groups of pigs were evaluated (slaughter pigs, pigs in areas of recent outbreaks, *etcetera*) and different assays were used. For acute disease, it is expected that one will detect ASFV nucleic acid in the blood or tissues. There are multiple validated real-time PCR (qPCR) assays for surveillance, individual, and herd testing, some are commercial kits and some published methodologies [22]. Validation requirements are outlined by the World Organisation for Animal Health (WOAH) [23]. Numerous assays have been developed with the ability to detect across genotypes as they target a highly conserved VP72 gene [24–28] and they are known for having a high level of sensitivity and specificity [22]. Yet, qPCR and PCR can only detect virus if it is present at appropriate levels in the tissues or blood. Detection using molecular techniques can be difficult and serology is needed animals that have survived acute disease, those with chronic disease [29], or with infections from attenuated viruses [30]. Antibodies against ASFV last for a very long time and can be reliably detected in pigs that were infected and survived ASFV. Most WOAH-approved ELISA tests target the p72 protein and up until 2019, the most widely used test was the Ingezim PPA Compaq K3 blocking ELISA, although multiple tests exist and there is WOAH guidance for use of an “in-house” ELISA [22, 31]. The manufacturer for the Ingezim ELISA reported a sensitivity of 99%, with 100% specificity [32], but other studies found that false positives did occur [33, 34]. WOAH recommends confirmation of ELISA positive results, particularly if there is any concern regarding sample quality [31].

There have been a wide number of studies in Uganda with varying results and ten years since a year-long assessment was done. Given the absence of a surveillance or monitoring system, it is critical to report on studies that can provide insight into the distribution, level, and timing of disease in the country. This study aimed to better ascertain the presence of ASFV in abattoir pigs from the greater Kampala metropolitan area while using a validated qPCR assay from a WOAH ASF reference laboratory and a WOAH-recommended commercial assay for serology. This study was the largest scale assessment of pigs for ASFV to date and aimed to provide a comprehensive diagnostic, spatial, and temporal assessment of ASFV seroprevalence and prevalence among pigs sampled at multiple abattoirs in the greater Kampala metropolitan area.

## Results

### Descriptive summary of sampled pigs

In total 1334 pigs were sampled, the majority were European breed pigs (56.2%; 750). A large number of pigs sampled were sourced from farms with < 11 pigs (1257; 94.2%). Most pigs were at the abattoir for one day (568; 42.6%) or two to five days (459; 34.4%). Pigs were reported to be healthy at purchase (97.8%; 1305) and after purchase (98.1%; 1308) (See Table 1).

**Table 1:** Descriptive summary of pigs sampled at abattoirs in the greater Kampala metropolitan area from May 2021 through June 2022

n = 1334	#	%
**Sex**		
Male	598	44.8
Female	729	54.6
Unknown	7	0.5
**Pig Type**		
Cross-bred	352	26.4
European	750	56.2
Local	201	15.1
Unknown	31	2.3
**Pig source**		
Farm (1–3 pigs)	550	41.2
Farm (4–11 pigs)	246	18.4
Farm (> 11 pigs)	461	34.6
Market	21	1.6
Unknown^*^	56	4.2
**Pig Heath at Purchase**		
Healthy	1305	97.8
Sick	11	0.8
Unknown	18	1.3
**Pig Health after Purchase**		
Healthy	1308	98.1
Sick	15	1.1
Unknown	11	0.8
**Duration of stay abattoirs**		
Same day	83	6.2
One day	568	42.6
2–5 days	459	34.4
Week	21	1.6
Unknown	203	15.2

*One trader reported purchasing the pig from a farmer, but the size of the farm was unknown

### ASFV nucleic acid and antibody detection

ASFV nucleic acid detection and serology results, presented for each sample type, are summarized in Table 2. For nucleic acid detection, blood had the lowest rate of detection (15.3%; 201/1316) and tonsil had the highest (38.0%, 474/1247). Spleen samples had the lowest median cycle threshold (Ct) value (17.359), and tonsils had the highest (25.33). The 95% confidence intervals (95%CI) overlapped for the percent positivity between lymph nodes (34.8–40.3%) and tonsils (35.4% and 40.7%), suggesting comparable findings,. When the pig-level overall status was evaluated (any of the sample types were positive), a 59.5% (95%CI: 56.9%, 62.1%) positivity rate was detected.

As for antibody detection, the screening ELISA detected 4 out of 1323 (0.3%) positive serum samples. Only two of the four (50%) screening positive samples were positive on the secondary ELISA; this resulted in a 0.15% seropositivity overall. All screened and confirmed seropositive samples were ASFV qPCR positive. Of the two pigs positive on both ELISA assays, one had mild signs of disease commensurate with ASFV infection: mild hemorrhage of lymph nodes, the kidney, and spleen. This sample was collected in March 2022. The other positive serologic sample came from a pig with no clinical signs or pathologic lesions and the sample was collected in July 2021. Of the two pigs positive only on the Ingenasa ASFV ELISA, one had clinical and pathologic signs of acute disease with erythema around the ears, abdomen, and flank as well as enlarged and hemorrhagic lymph nodes and spleen and a hemorrhagic kidney. These pigs were sampled in May and June of 2021.

**Table 2:** Diagnostic African swine fever virus summary of pigs sampled at Kampala area abattoirs, 2021–2022

	ASFV real-time PCR results					
Sample type	# positive	%	Total # tested	95% confidence interval	Median Ct Value	Range
Blood	201	15.3	1316	13.4, 17.3	24.807	16.231, 39.957
Spleen	359	31.5	1254	29.0, 34.1	17.359	12.918, 39.756
Lymph node	453	37.5	1208	34.8, 40.3	22.806	14.37, 38.997
Tonsil	474	38.0	1247	35.4, 40.7	25.33	15.4, 38.395
Overall pig status	794	59.5	1334	56.9, 62.1	--	--
	**ASFV serology screening results**					
**Sample type**	**# positive**	**%**	**Total # tested**	**95% confidence interval**		
Serum (screening)	4	0.3	1323	0.1, 0.8	--	--
Serum (confirmatory)	2	50	4	15.0, 85.0	--	--
Serum overall	2	0.15	1323	0.003, 0.6	--	--

Agreement between sample types was also evaluated for nucleic acid detection (Table 3). Percent agreement ranged from 65.3% (spleen vs. tonsil) to 75.3% (blood vs. spleen), and the overall agreement was 70.8%. The Brennan and Prediger (B&P) kappa was consistently higher than the traditional Cohen kappa. The greatest agreement was found between blood and spleen samples with a Cohen kappa of 0.33 and a B&P kappa of 0.505, fair and moderate agreement, respectively.

**Table 3:** Agreement of African swine fever virus qPCR results for pigs sampled at Kampala area abattoirs

		Cohen		Brennan & Prediger	
Sample comparisons	Percent agreement	Kappa	95% confidence interval	Kappa	95% confidence interval
All sample types	70.8%	0.32	0.29, 0.36	0.42	0.38, 0.45
Blood vs. spleen	75.3%	0.33	0.28, 0.39	0.505	0.46, 0.55
Blood vs. lymph node	71.2%	0.31	0.25, 0.35	0.43	0.37, 0.48
Blood vs. tonsil	72.3%	0.335	0.29, 0.38	0.45	0.395, 0.50
Spleen vs. lymph node	67.3%	0.28	0.22, 0.34	0.345	0.29, 0.40
Spleen vs. tonsil	65.3%	0.24	0.18, 0.30	0.31	0.25, 0.36
Lymph node vs. tonsil	71.0%	0.38	0.33, 0.44	0.42	0.37, 0.47

**Fig. 1:**
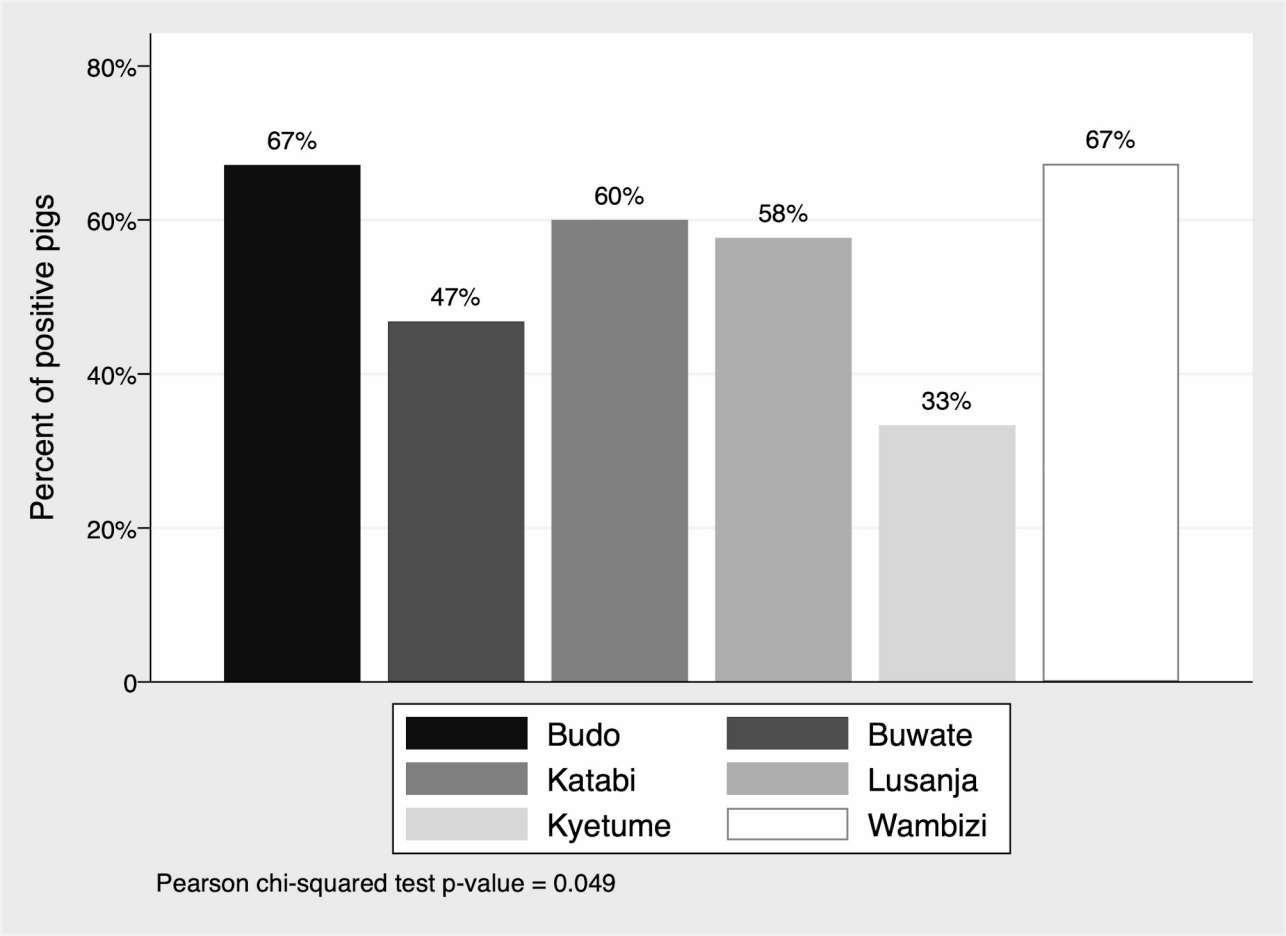
Percent positivity pigs by abattoir from which they were sampled in the Kampala area. A pig was positive if any tissue had African swine fever virus nucleic acid detected

There were also differences in the percent positivity of pigs sampled by abattoir (See Fig. 1). Budo and Wambizi had the highest percent positivity of 67% and Kyetume had the lowest (33%). There was a significant association between abattoir and ASFV status (p-value = 0.049). A spatial overview of where pigs from each abattoir originated from is available in Supplementary Tables S1 and Figures S1 to S6. The greatest capture area was from the Wambizi abattoir with 37 different districts represented in the pigs sampled with a median of five and a range of one to 101 pigs from different districts. In contrast, Kyetume only had two districts represented in the pigs sampled with 96.8% (60) coming from the Mukono district where Kyetume is located.

### Spatiotemporal analysis of nucleic acid detection results

**Fig. 2:**
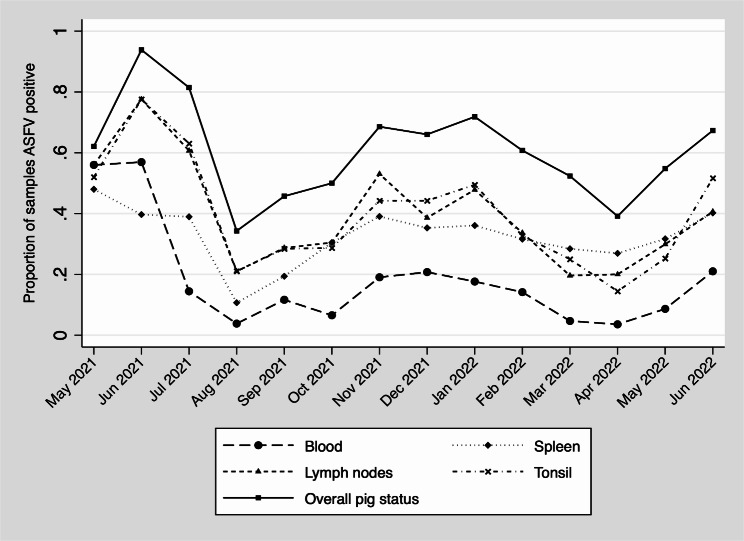
Proportion of African swine fever virus positive of samples from pigs slaughtered in Kampala. A statistical analysis comparing ASFV results by month of the year between May 2021 and June 2022 using a Pearson chi-squared had a p-value < 0.001 for all tissue types and the overall pig status

Temporal patterns of ASFV can be seen in Fig. 2 andTable 4. There was a statistical association between ASFV status and the month of the year for all tissue types and the overall pig ASFV status (all p-values < 0.001). There was also a statistically significant association (p-value<0.001) between ASFV status and whether the pig was sampled in the wet (March to May and September to November) or dry season. The highest periods of ASFV detection were observed for samples collected between November and February and May through July.

**Table 4:** Comparison of pigs by ASFV status and season sampled at Kampala area abattoirs

	ASFV positive pigs	Total pigs	P-value
Season	# (%)	#	< 0.001
Rainy season	453 (66.2)	684	
Dry season	341 (52.5)	650	
	794 (40.5)	1334	

The rainy season is defined as March through May and September through November [35]

**Fig. 3:**
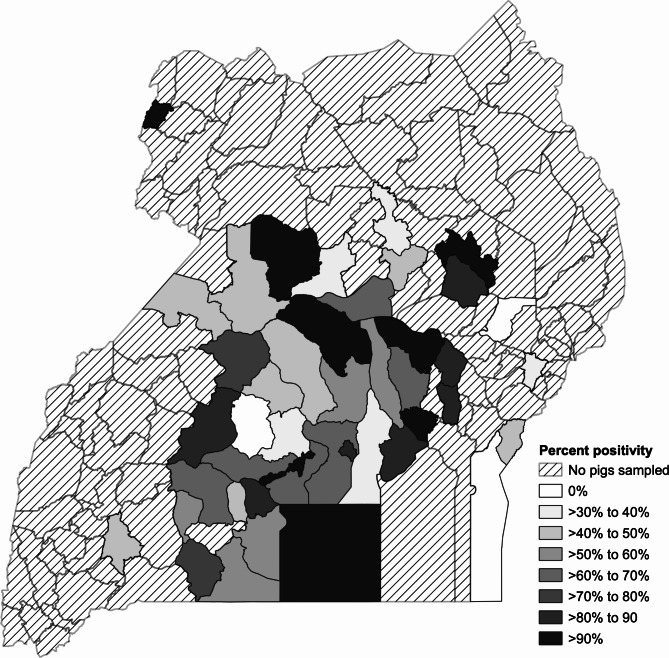
African swine fever percent positivity of pigs sampled from Kampala area abattoirs district of origin. Testing was completed using a real-time PCR assay for detection of nucleic acid. A pig was considered positive if any of the tissue samples from that pig were positive. Pigs with unknown districts of origin were excluded but had a percent positivity of 50%

The pigs sampled in this study originated from 44 (32.4%) districts out of 136 districts on the map of Uganda (See Fig. 3). Sample sizes in the districts ranged from 1 to 359 pigs sampled with a median sample size of 9 (interquartile range: 4.5, 25.5) (Supplementary Table S2). The median percent positivity among all districts was 60.7% (interquartile range: 50%, 87.1%). There were only three districts where all pigs were negative on qPCR; they only had one to seven pigs sampled. There were eight districts that had a > 90% positivity and six of them had 100% percent positivity. These districts with 100% positivity only had one to six pigs sampled (See Fig. 3).

## Discussion

This study provides one of the more comprehensive assessments of ASFV presence and distribution in Uganda from pigs sampled at multiple Kampala metropolitan area abattoirs. Serum was evaluated for antibodies and multiple tissue types and blood were tested for ASFV nucleic acid using qPCR; they were all validated tests and procedures. Previous studies with similar efforts, but smaller scope, had varying results. A longitudinal analysis in Masaka and Rakai districts, located in the eastern portion of southwest Uganda, in 2010 and 2011, had no pigs with antibodies. Serology was performed using two commercial kits produced by the Ingezim PPA Compac 1.1 PPA K3 blocking ELISA with testing of positive result completed using an indirect ELISA from Svanovir [21]. Farmers reported a robust incidence of outbreaks. Yet, only three pigs (0.4%) had a positive blood sample when tested using qPCR. This study found similar seropositivity results (0.15%), in abattoir pigs, but found a high overall ASFV nucleic acid percent positivity in abattoir pigs (59.5%) compared to pigs sampled at the farm. A cross-sectional and longitudinal study published in 2017 was also conducted along the Kenya-Uganda border. For serology, the Ingezim PPA Compac 1.1 PPA K3 was again used, for viral nucleic acid detection a conventional PCR and three qPCRs were used. All pigs in the cross-sectional study (1107) were negative on qPCR/PCR, and 5 pigs out of the starting sample of 232 were positive during the entire longitudinal study. Only one pig was seropositive. A small sample of pigs at the local abattoirs (n = 28) were also tested and 15 (53.6%) were positive using qPCR and conventional PCR [19]. This study found similar results to the 2010 and 2011 study among pigs sampled at the farm, but also noted that abattoir sampled pigs had a higher positivity. Their abattoir positivity (53.6%) was similar to this study (59.5%). Finally, a study from 2013 conducted both a year-long abattoir survey and a district level survey in Western and Central Uganda. They visited one Kampala area abattoir twice a month for one year and collected blood samples for ASFV analysis using an in-house ELISA following WOAH’s Manual of Diagnostic Test procedures and a conventional PCR. They detected very high seroprevalence of 52.96% and an 11.5% ASFV nucleic acid prevalence in abattoir pigs and a 53.95% seroprevalence and 11.5% ASFV nucleic acid prevalence in the districts [20]. Those results differed substantially from this work where the seroprevalence was 0.15% and the percent positivity of pigs for ASFV nucleic acid was 59.5%.

The differences between studies could reflect changes over time but may also reflect diagnostic improvements as well. qPCR assays have been shown to have greater sensitivity relative to conventional assays and, with the use of internal positive controls, greater quality assurance [24, 26, 36]. Confirmatory serologic testing is important when testing pigs for exposure to ASFV. Despite the high sensitivity of the screening assay used in this study, it has been shown in previous work to produce false positive results [33]. We only detected a handful of pigs with antibodies against ASFV, one with clinical signs and one without. This result suggests that there are not many pigs that survive disease or are infected with low virulent strains, which may cause subclinical or chronic disease, although further study is needed.

Use of abattoirs for ASFV research likely reveals the significant number of ASFV cases in the country, could be a method to detect areas with ASFV outbreaks, and provides a population to monitor areas of ASFV activity that is more efficient than monitoring on-farm pigs. Monitoring could be targeted at abattoirs with the highest percent positivity among pigs (Wambizi and Budo, Fig. 1), the greatest catchment area (Wambizi; Supplementary Figure S6), or the most unique catchment area (Lusanja; Supplementary Figure S5). Abattoir surveillance will not provide early detection of outbreaks though. In addition, the ASFV nucleic acid percent positivity calculated from such samples is likely inflated compared to the true national district-level prevalence. Based on the results in this study, qPCR would be needed for such a program. The use of serology to screen for cases at abattoirs would not be effective, as pigs either do not survive infection or are infected but pre-clinical and without a detectable level of antibodies. The laboratory capability for qPCR testing is present at the National Animal Disease Diagnostic and Epidemiology Center in Entebbe, Uganda and at the Central Diagnostic Laboratory in the College of Veterinary Medicine, Animal Resources and Biosecurity at Makerere University in Kampala, Uganda. The cost of the assays would have to be considered against the cost of unmanaged outbreaks before implementing such a program.

The very high percent positivity for ASFV nucleic acid found in this study could be related to the sampling methodology of using abattoir pigs. These animals may be more likely to come from active outbreaks and would overestimate the prevalence. It is well-documented that Ugandan farmers will sell off pigs in the face of an ASFV outbreak [37–39]. Further, exposure to virus from transport vehicles that were not cleaned and disinfected as well as from other pigs contacted during transport can also occur [37]. This may lead to increased ASFV infections among abattoir pigs relative to the district pig population. This is most feasible among pigs that are held at the abattoir for a few days to a week before they are slaughtered, which was reported to occur in this study. Therefore, it is not likely that this study provided a reliable prevalence estimate for the districts. Yet this information is still useful. It is likely abattoirs are catchment areas for pigs sold in response to ASFV infections. In a study among those involved in the Ugandan pig trade with small holders, panic sales of pigs were believed to be a risky practice [37], and modeling has provided evidence that this act increases the risk of disease spread as well [40]. The farmers in this study were reported to be small-scale producers (< 11 pigs, the majority had 1–3), similar to the study just cited, and they may also respond to ASFV infections in their areas by selling pigs, which would spread disease to new areas. Identifying ASFV infected pigs and current areas of circulating virus through abattoir surveillance could assist in disease control by reducing disease spread associated with panic sales, which would include pig-associated transmission and transmission from infected pork and pork products [41–43].

Surveillance at abattoirs could be used to identify ASFV infected pigs and to trace them back to infected areas that sold them in response to the disease. The spatial assessment provides an understanding of the catchment area of the greater Kampala metropolitan area abattoirs, and this catchment area overlaps with the previously published pig density distribution maps in Uganda [44–46]. Therefore, the greater Kampala metropolitan area abattoirs likely represent a large portion of the Ugandan pig population. The districts from which no pigs were sampled during this study may use local abattoirs and are also reported areas of low pig density [44–46]. Other studies have done work along the Kenya-Uganda border [6, 19] and there were no pigs sampled from that region in this study as well. Therefore, studies are needed to identify ideal locations for convenient and efficient ASFV surveillance activities in districts not represented in this study. This should include assessments on the use of serology.

Since molecular diagnostic testing, specifically qPCR, should be a critical component of surveillance at abattoirs, agreement for ASFV nucleic acid detection between sample types should be considered. Agreement between sample types varied between the traditional Cohen’s kappa and bias and prevalence adjusted B&P kappa. This suggests that disease prevalence and, potentially, diagnostic sensitivity of the samples type impacts agreement. ASFV nucleic acid percent positivity of pigs, as determined by the tonsil and lymph node samples, was higher and had overlapping 95%CIs, while spleen and blood had lower percent positivity with 95%CIs that did not overlap with any other sample type. In pigs naturally infected with ASFV, the tonsils are the first site of viral detection, along with lymph nodes of the head and neck [2, 47]. These sample types may be more reliable for early detection from sell-offs. Multiple sample types may need to be tested, and use of blood alone may miss infected pigs.

Temporal patterns of ASFV detection reflect that disease is more often seen in dry seasons both based on monthly trends and a comparison of ASFV status of pigs and the season (rainy or dry) that they were sampled. ASFV detection was highest from May to June and November to February, periods outside of the rainy season. The primary rainy seasons have been defined as occurring from March through May and a secondary season is seen in September through November. Regular rain is also reported between June and August, although these months were not characterized as a rainfall band [35]. This association between seasonality and ASFV detection should be further evaluated over multiple years to ensure that this finding is repeatable, and, if it is repeatable, to determine the reason for increased ASFV during drier periods. Further, rainy season patterns are changing because of climate change [35]. Such changes to rain patterns may lead to different temporal patterns related to ASFV transmission and future control of this virus in Uganda and East Africa may rely on better understanding this seasonal relationship better.

There were a few limitations to this study. First was the purposive selection of abattoir. They were selected based on information from the abattoir managers as to the catchment area and annual slaughter rates. This allowed for the study to represent the greatest number of pigs that are sent to the greater Kampala metropolitan area for slaughter. Yet, many districts had very low numbers of pigs sampled, limiting some of the interpretation and ability to provide statistical analysis. We were also limited in that we were not able to use a WOAH confirmatory serologic test due to lack of availability (immune blotting assay) and an inability to culture virus (needed for use of the indirect immune-peroxidase assay). Instead, we used testing in series with another commercial ELISA with a different mechanism of action and antigen targets to maximize specificity, which is an appropriate approach. These results represent pigs sent to these abattoirs and the discussion highlights how abattoir sampling can be used for greater control programs in the country, but do not relate to pigs raised and consumed in local villages. Clinical and pathologic signs data were collected, but the scale and scope of that information is too large to be adequately summarized here and is being submitted for publication separately.

## Conclusions

This study revealed a very high level of ASFV infected pigs at abattoirs in Kampala but a very low level of pigs with ASFV antibodies. This is critical as these pigs are likely to contribute to the transmission cycle in the country as pork and pork products are risk factors for transmission to pigs when restaurant or household waste are fed to pigs. Further, abattoir sampling can be used to detect outbreaks in district of origin, and abattoirs in Kampala have a catchment area that covers most of the highest pig density areas in the country. This study suggests there may be a seasonality of ASFV infections that should be monitored as climate change alters patterns of rain in the country.

## Materials and methods

### Sampling methodology and collection

**Fig. 4:**
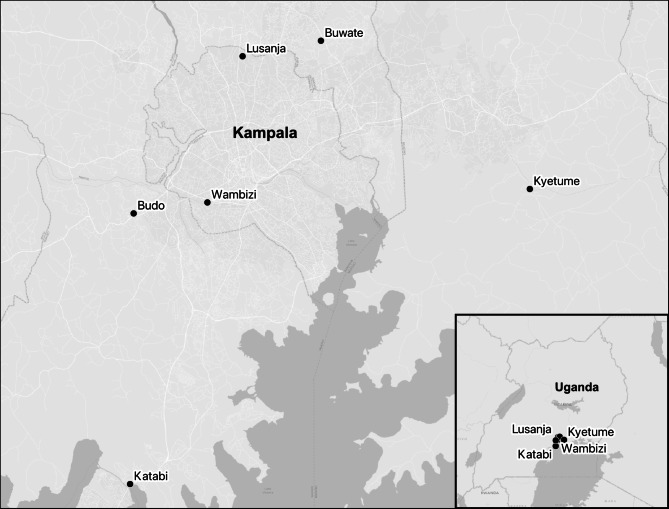
Ugandan map showing six abattoirs in the greater Kampala metropolitan area where pigs were sampled

Pigs were sampled from six abattoirs in the greater Kampala metropolitan area from May 2021 through June 2022. The abattoirs were Lusanja, Buwate, Kyetume, Budo, Katabi, and Wambizi (Fig. 4). A stratified, systematic sampling approach was used by weighting sample sizes at each abattoir by the average annual slaughter rates reported by each site (Table 5). If an odd number of samples were needed per month, the sample number was rounded up to the next even number and a minimum sampling number per visit was set at four to ensure proper application of the systematic sampling described below (Table 5). Two to four days were randomly selected each month for sampling at each abattoir. Systematic sampling was used on the day of slaughter to representatively sample pigs at the site until the designated sample sizes were reached. Pigs were sampled regardless of health status to ensure any low virulent, chronic, or inapparent ASFV strains could be captured if present. The sample size needed to detect the expected prevalence of 11.5% [20] with 95% confidence and 5% error was 157 pigs (openepi.com; Accessed July 2018). Of those 157 pigs, eighteen would be positive given the expected prevalence. Therefore, among the six abattoirs, 1200 pigs were sampled to ensure there were over 100 positive pigs. The intent of capturing at least 100 pigs was to have a reasonable number to assess characteristics while considering logistical constraints. This sample size also allowed for detection of any prevalence with > 99.9% confidence and 5% error.

**Table 5:** Sampling design for abattoirs in the greater Kampala metropolitan area, May 2021 through June 2022

Abattoir	Pigs slaughtered per year	Pigs sampled per month	# visits per month	Pigs sampled per visit	Total pigs sampled
Lusanja	43,200	36	4	9	443
Wambizi	36,000	32	4	8	396
Katabi	19,200	16	2	8	195
Budo	10,800	10	2	5	135
Buwate	9000	8	2	4	103
Kyetume	7200	8	2	4	62^*^

^*^ Sample sizes were not met for Kyetume due to COVID-19 limitations and economic pressures that reduced slaughter numbers in 2021

For each pig sampled, pig traders provided their district of origin along with other meta-data. Pig traders were provided 12,500 Ugandan shilling ($3–4 USD) for each pig they provided information on and allowed sampling of to reimburse them for the extra time taken. Antemortem blood samples were collected using a 21-gauge needle, whole blood was collected into a 10 ml EDTA tube (Becton, Dickinson, and Company, Franklin Lakes, New Jersey, USA) and serum into a 10 ml clotting tube (Becton, Dickinson, and Company, Franklin Lakes, New Jersey, USA). Post-mortem, tonsil, lymph nodes (submandibular, renal, and gastro-hepatic), and spleen samples were collected using separate gloves, forceps, and scalpels between each tissue type to prevent cross-contamination. Each tissue type was transported in a separate tissue collection bag, placed in a cold box for transport, and stored at -20°C until processing and extraction occurred.

### Diagnostic testing

All tissue processing and testing was completed following the US Department of Agriculture’s (USDA) Foreign Animal Disease Diagnostic Laboratory’s (FADDL) standard operating procedures (SOPs). FADDL is designated as an ASF Reference Center by WOAH (https://www.woah.org/en/what-we-offer/expertise-network/reference-laboratories/#ui-id-3, Accessed October 2, 2023).

Preparation of the samples was as follows. Each whole blood sample was diluted 1:1 with 1X phosphate buffered saline (PBS) (Thermo Fisher Scientific, Waltham, Massachusetts, USA). For tissue samples, 1 g of the tissue was weighed out, washed in 1X PBS, and then homogenized in a stomacher bag using the Stomacher® 80 Biomaster (Seward Ltd, West Sussex, United Kingdom). The homogenized tissue was combined with 9 ml 1X PBS and centrifuged at 1000 x g for 10 min. Approximately 1.5 mL of the supernatant was collected and stored at -20°C until it was used for viral DNA extraction.

Total DNA extraction was performed using the Qiagen DNeasy tissue and blood kit (Qiagen, Hilden, Germany), following the US Department of Agriculture’s (USDA) Foreign Animal Disease Diagnostic Laboratory’s (FADDL) standard operating procedures (SOPs), which follow the manufacturer’s instructions [48]. The 1:1 blood-to-PBS dilution was performed as per the manufacturer’s and FADDL SOP’s instructions to maximize the extracted DNA [49]. The real-time PCR assay used was previously described [25] and the FADDL SOP was again followed [50]. The TaqMan® Fast Virus 1-Step Master Mix (Thermo Fisher Scientific, Waltham, Massachusetts USA) along with the forward primer of 5’-CCTCGGCGAGCGCTTTATCAC-3’, reverse primer of 5’-GGAAACTCATTCACCAAATCCTT-3’, and probe of FAM-CGATGCAAGCTTTAT-MGB/NFQ (Eurofin Genomic, Munich, Germany) were used in the qPCR procedure. The VetMax Xeno DNA internal positive control (IPC) (Thermo Fisher Scientific, Waltham, Massachusetts USA) was used during the DNA extraction procedures and the VetMax Xeno IPC LIZ Assay (Thermo Fisher Scientific, Waltham, Massachusetts USA) was used during the qPCR. This was done for each individual sample following FADDL SOPs. The qPCR assay was run on a QuantStudio 5 thermocycler (Thermo Fisher Scientific, Waltham, Massachusetts USA). A positive result was any sample with a cycle threshold < 40.

Screening for ASFV antibodies in serum was performed using the INgezim PPA COMPAC ASF ELISA kit PPA.3 (Gold Standard Diagnostics, Madrid, Spain). This kit was validated and used by the USDA FADDL. The FADDL SOP was followed [51], which also followed manufacturer’s instructions. This is a competitive ELISA targeted against the P72 protein with a manufacturer reported sensitivity and specificity of 99% and 100% [32], although false positives have also been reported [33]. The optical density was read at a wavelength of 450 nm (OD_450_) using a Multiscan FC ELISA reader (Thermo Scientific, Waltham Massachusetts, USA). The following were the cut-off calculations using the negative control (NC) and positive control (PC):

$${\text{Positive cut-off = NC-}}\left[ {\left( {{\text{NC-PC}}} \right){\text{ * 0}}{\text{.5}}} \right]$$

$${\text{Negative cut-off = NC-}}\left[ {\left( {{\text{NC-PC}}} \right){\text{ * 0}}{\text{.4}}} \right]$$

The mean OD_450_ for the negative control had to be a minimum of four times higher than the positive controls OD_450_ for the test to be valid. Samples with a mean OD_450_ between the cut-off values were doubtful, those with values greater than the positive cut-off were positive and those with values below the negative cut-off were negative.

A WOAH approved confirmatory test was unavailable commercially or from reference laboratories and the granting entity did not allow for culture of the pathogen, therefore we were not able to perform any WOAH approved confirmatory assays . Instead, the IDScreen® (Innovative Diagnostics, Montpellier, France) indirect ELISA that targets antibodies against P32, P62 and P72 was used for confirmation of positive screening results through testing in series [52]. Secondary testing with an indirect assay has also been reported previously [21]. This assay had a different mechanism of action (indirect ELISA), targeted additional antigenic proteins, and has a 100% specificity when doubtful results are considered negative. The optical density of samples was read at 450 nm (OD450) on a Multiscan FC ELISA reader (Thermo Scientific, Waltham Massachusetts, USA). Sample to positive percentages (S/P) were calculated with S/P ≤ 30% being classified as negative, S/P ≥ 40% as positive, and between 30% and 40% as doubtful.

### Data management and statistical analysis

The ELISA results were reviewed for validity of the negative and positive control before data entry. The qPCR results were reviewed for validity of internal positive controls, extraction controls and amplification controls. Data were then entered into spreadsheets using Excel version 16.71 (Microsoft, Redmond, Washington, USA) and reviewed for duplicate entries and missing results. For qPCR results, occasionally tissues were inadvertently tested in duplicate as there were multiple vials of the same tissue stored. These duplicate test entries were assessed for comparability of cycle threshold values (Ct). If the duplicate results were within three Ct values of one another, the first sample tested, and its Ct value was kept. If the results were > 3 Ct values apart the results were discarded. There were no duplicate entries for the ELISA results. Untested and discarded duplicate samples were not tested as the funding agency stopped work on all projects in Uganda for political reasons in the spring of 2023. In total, out of 1334 pigs sampled, 1323 (99.2%) serum samples, 1316 (98.7%) blood samples, 1254 (94.0%) spleen samples, 1208 (90.1%) lymph nodes, and 1247 (93.5%) tonsils were tested. All available serum samples that were not hemolyzed and had enough volume were tested.

Frequency and percentages were calculated for the pig descriptive statistics. Frequency and percentage positivity values were calculated for each sample type and for the overall pig status. A pig was seropositive if both ELISA results were positive, and overall pig status refers to all pigs with at least one qPCR positive sample type (blood, lymph node, spleen, or tonsil). For the percentage positive values the Agresti-Coull 95% confidence intervals [53, 54], which uses estimated intervals and is best with sample sizes > 40, were calculated along with the median and range of Ct values for each tissue type. Cohen [55, 56] and Prediger-Brennan kappa statistics were calculated to compare agreement of results across tissue types for qPCR results. The latter controls for prevalence and bias [57, 58]. Kappa scores were described by level agreement using the methodology described by Landis and Koch [59]. Frequencies and percentages were calculated for pig ASFV cases by dry and rainy season as previously described [35] add abattoir and a Pearson’s chi-squared tests was performed to evaluate that association. A connected line graph was built to depict annual proportional trends of infection by month by tissue type and pig as well and a bar graph was used to depict overall pig percent positivity by abattoir. Medians and interquartile ranges (IQR) were calculated for sample sizes and percent positivity within the districts. Stata 16.1 IC (Stata Corp, College Station, Texas, USA) was used for all statistical calculations.

Finally, maps were created to show the geographic distribution and prevalence of ASFV among the pigs sampled. Mapping of abattoir locations used GPS coordinates collected via Google Maps (Google, Mountain View, California, USA) at each abattoir and ESRI maps embedded in QGIS Firenze version 3.28.1 (qgis.org). Shape files for Ugandan districts were obtained from the United Nations High Commissioner for Refugees Operational Data Portal (https://data.unhcr.org/en/documents/details/83043; Accessed March 30, 2023). The shape files were published on November 17, 2020. These shape files were linked to overall pig percent positivity data calculated for each district regardless of sample size in the district and then mapped.

### Electronic supplementary material

Below is the link to the electronic supplementary material.

Supplementary Material 1

## Data Availability

Data is available upon reasonable request to the corresponding author.
