# Supplementary material for: Spatiotemporal description of African swine fever virus nucleic acid and antibodies detected in pigs sampled at abattoirs in the greater Kampala metropolitan area, Uganda from May 2021 through June 2022
Source: Porcine Health Manag. 2023 Nov 2;9:51. doi: 10.1186/s40813-023-00345-7 (PMC10623799; doi:10.1186/s40813-023-00345-7)
Supplement: Supplementary file 1 — Supplementary Material 1 [file 40813_2023_345_MOESM1_ESM.docx]

Supplementary Files

Table S1: Distribution of pigs sampled based on district of origin by abattoir in the greater Kampala metropolitan area, May 2021 through June 2022.

| **Abattoir** | **District** | **Number of pigs sampled per district** | **Percentage of pigs sampled per district** |
| --- | --- | --- | --- |
| Budo | Buyende | 5 | 3.7 |
| Budo | Gomba | 6 | 4.4 |
| Budo | Jinja | 1 | 0.7 |
| Budo | Kaliro | 3 | 2.2 |
| Budo | Kalungu | 3 | 2.2 |
| Budo | Kamuli | 8 | 5.9 |
| Budo | Masaka | 22 | 16.3 |
| Budo | Mpigi | 28 | 20.7 |
| Budo | Mubende | 4 | 3 |
| Budo | Wakiso | 54 | 40 |
| Budo | Unknown | 1 | 0.7 |
| Buwate | Amolatar | 11 | 10.7 |
| Buwate | Buikwe | 4 | 3.9 |
| Buwate | Dokolo | 4 | 3.9 |
| Buwate | Iganga | 2 | 1.9 |
| Buwate | Jinja | 2 | 1.9 |
| Buwate | Kayunga | 5 | 4.9 |
| Buwate | Kiboga | 16 | 15.5 |
| Buwate | Luwero | 4 | 3.9 |
| Buwate | Masindi | 4 | 3.9 |
| Buwate | Mbale | 1 | 1 |
| Buwate | Mpigi | 1 | 1 |
| Buwate | Mukono | 3 | 2.9 |
| Buwate | Nakaseke | 5 | 4.9 |
| Buwate | Wakiso | 25 | 24.3 |
| Buwate | Unknown | 16 | 15.5 |
| Katabi | Apac | 3 | 1.5 |
| Katabi | Gomba | 1 | 0.5 |
| Katabi | Hoima | 4 | 2.1 |
| Katabi | Kamuli | 2 | 1 |
| Katabi | Kiboga | 8 | 4.1 |
| Katabi | Kyankwanzi | 8 | 4.1 |
| Katabi | Masaka | 4 | 2.1 |
| Katabi | Mityana | 2 | 1 |
| Katabi | Mpigi | 4 | 2.1 |
| Katabi | Wakiso | 156 | 80 |
| Katabi | Unknown | 3 | 1.5 |
| **Abattoir** | **District** | **Number of pigs sampled per district** | **Percentage of pigs sampled per district** |
| Kyetume | Mukono | 60 | 96.8 |
| Lusanja | Amolatar | 18 | 4.1 |
| Lusanja | Bukomansimbi | 14 | 3.2 |
| Lusanja | Dokolo | 2 | 0.5 |
| Lusanja | Gomba | 1 | 0.2 |
| Lusanja | Jinja | 2 | 0.5 |
| Lusanja | Kampala | 11 | 2.5 |
| Lusanja | Kamuli | 20 | 4.5 |
| Lusanja | Kayunga | 51 | 11.5 |
| Lusanja | Kumi | 7 | 1.6 |
| Lusanja | Kyotera | 15 | 3.4 |
| Lusanja | Luwero | 101 | 22.8 |
| Lusanja | Maracha | 1 | 0.2 |
| Lusanja | Masaka | 57 | 12.9 |
| Lusanja | Mbarara | 1 | 0.2 |
| Lusanja | Mityana | 2 | 0.5 |
| Lusanja | Mpigi | 8 | 1.8 |
| Lusanja | Mukono | 3 | 0.7 |
| Lusanja | Nakaseke | 26 | 5.9 |
| Lusanja | Nakasongola | 5 | 1.1 |
| Lusanja | Namayingo | 1 | 0.2 |
| Lusanja | Rakai | 2 | 0.5 |
| Lusanja | Wakiso | 82 | 18.5 |
| Lusanja | Unknown | 13 | 2.9 |
| Wambizi | Amolatar | 4 | 1 |
| Wambizi | Amuria | 1 | 0.3 |
| Wambizi | Bukomansimbi | 2 | 0.5 |
| Wambizi | Busia | 2 | 0.5 |
| Wambizi | Butambala | 1 | 0.3 |
| Wambizi | Buyende | 1 | 0.3 |
| Wambizi | Gomba | 12 | 3 |
| Wambizi | Iganga | 9 | 2.3 |
| Wambizi | Jinja | 6 | 1.5 |
| Wambizi | Kalangala | 5 | 1.3 |
| Wambizi | Kaliro | 6 | 1.5 |
| Wambizi | Kalungu | 5 | 1.3 |
| Wambizi | Kampala | 4 | 1 |
| Wambizi | Kamuli | 18 | 4.5 |
| Wambizi | Kasanda | 2 | 0.5 |
| Wambizi | Kayunga | 3 | 0.8 |
| Wambizi | Kiboga | 2 | 0.5 |
| **Abattoir** | **District** | **Number of pigs sampled per district** | **Percentage of pigs sampled per district** |
| Wambizi | Kyotera | 10 | 2.5 |
| Wambizi | Lira | 6 | 1.5 |
| Wambizi | Luwero | 2 | 0.5 |
| Wambizi | Lyantonde | 5 | 1.3 |
| Wambizi | Masaka | 101 | 25.5 |
| Wambizi | Masindi | 5 | 1.3 |
| Wambizi | Mbale | 2 | 0.5 |
| Wambizi | Mbarara | 1 | 0.3 |
| Wambizi | Mityana | 8 | 2 |
| Wambizi | Mpigi | 21 | 5.3 |
| Wambizi | Mubende | 10 | 2.5 |
| Wambizi | Mukono | 9 | 2.3 |
| Wambizi | Nakaseke | 1 | 0.3 |
| Wambizi | Nakasongola | 12 | 3 |
| Wambizi | Rakai | 7 | 1.8 |
| Wambizi | Soroti | 17 | 4.3 |
| Wambizi | Ssembabule | 26 | 6.6 |
| Wambizi | Wakiso | 42 | 10.6 |
| Wambizi | Unknown | 25 | 6.3 |


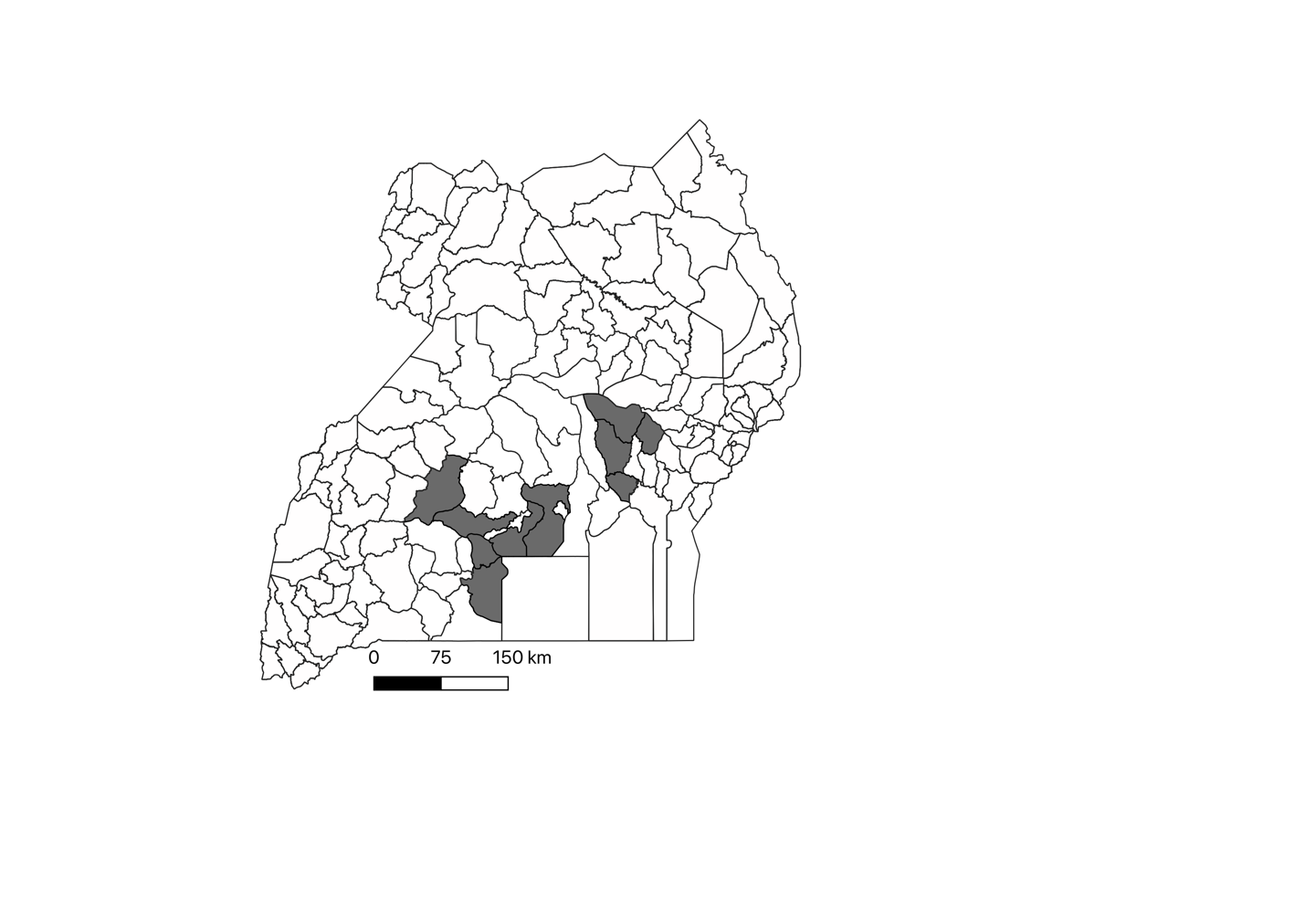


Figure S1: Distribution of Ugandan districts from where pigs sampled at the Budo abattoir from May 2021 through June 2022 originated.


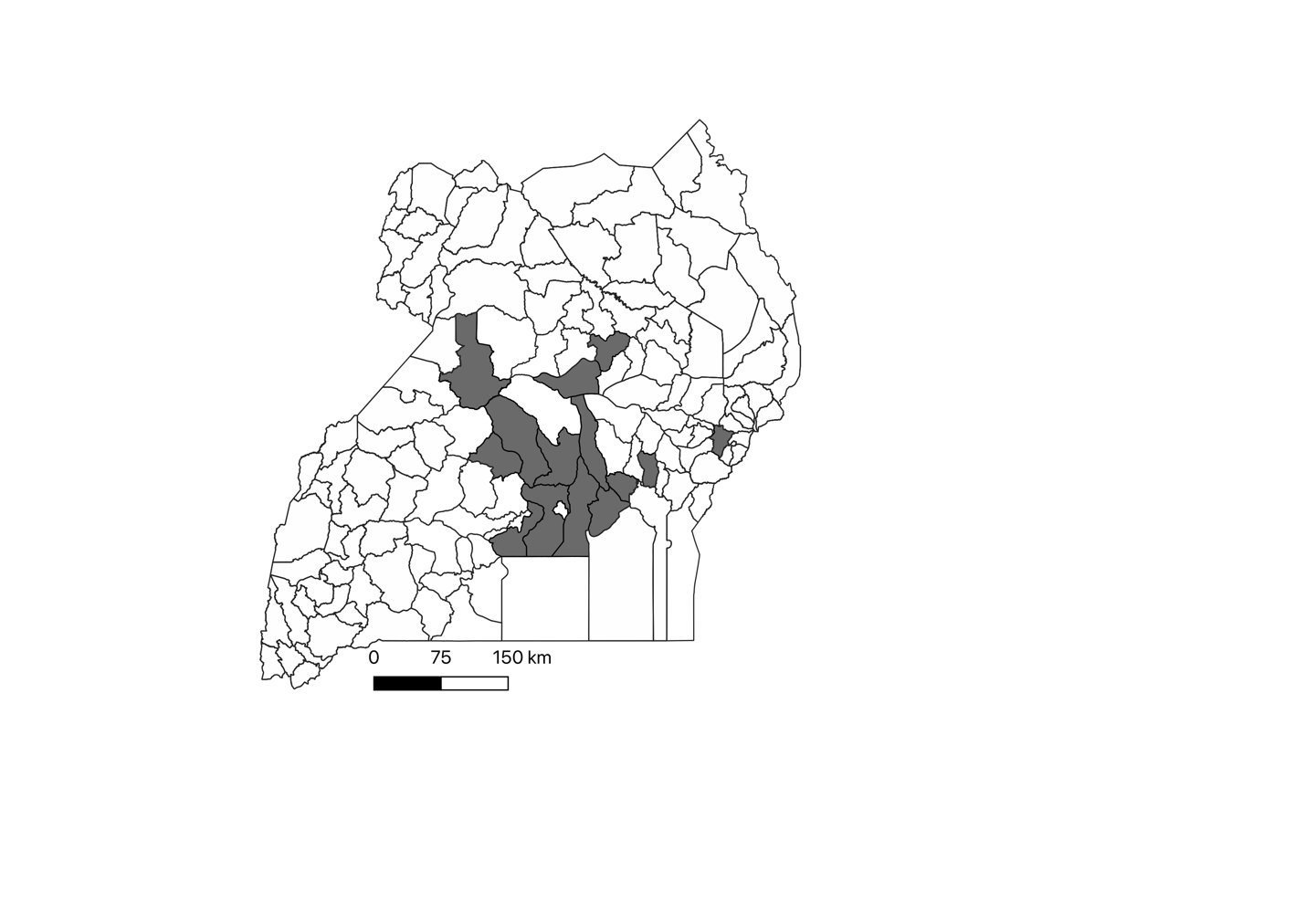


Figure S2: Distribution of Ugandan districts from where pigs sampled at the Buwate abattoir from May 2021 through June 2022 originated.


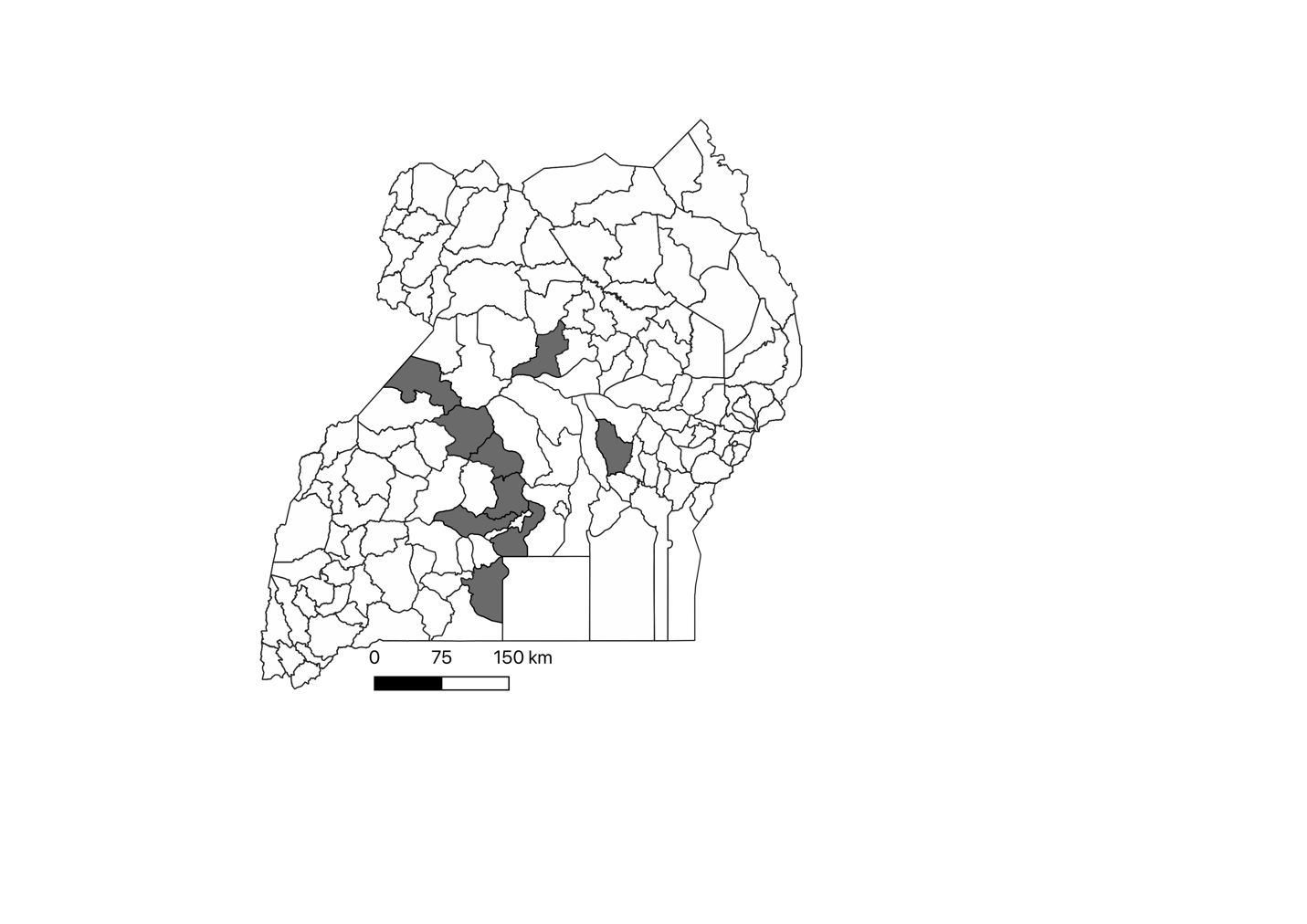


Figure S3: Distribution of Ugandan districts from where pigs sampled at the Katabi abattoir from May 2021 through June 2022 originated.


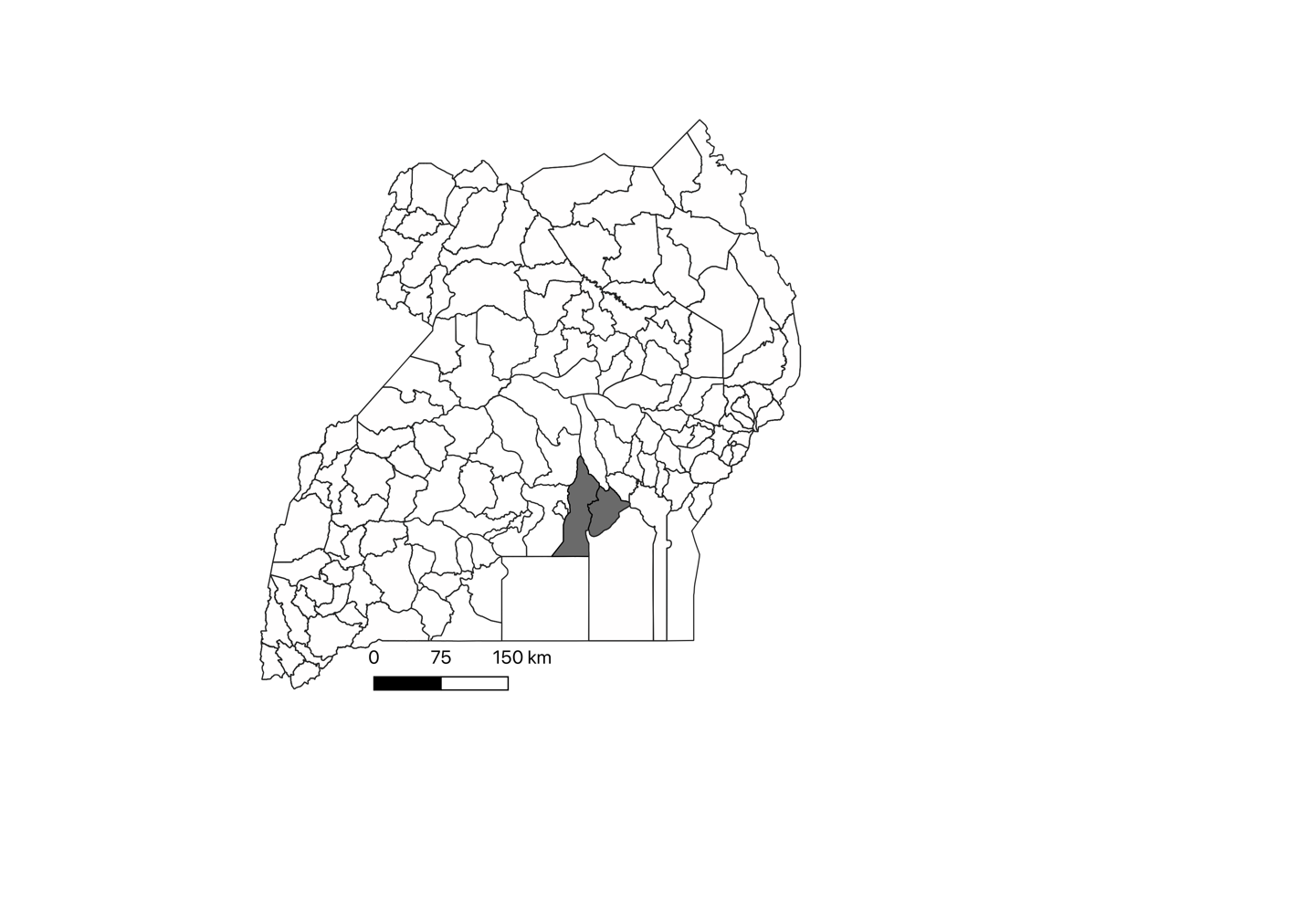


Figure S4: Distribution of Ugandan districts from where pigs sampled at the Kyetume abattoir from May 2021 through June 2022 originated.


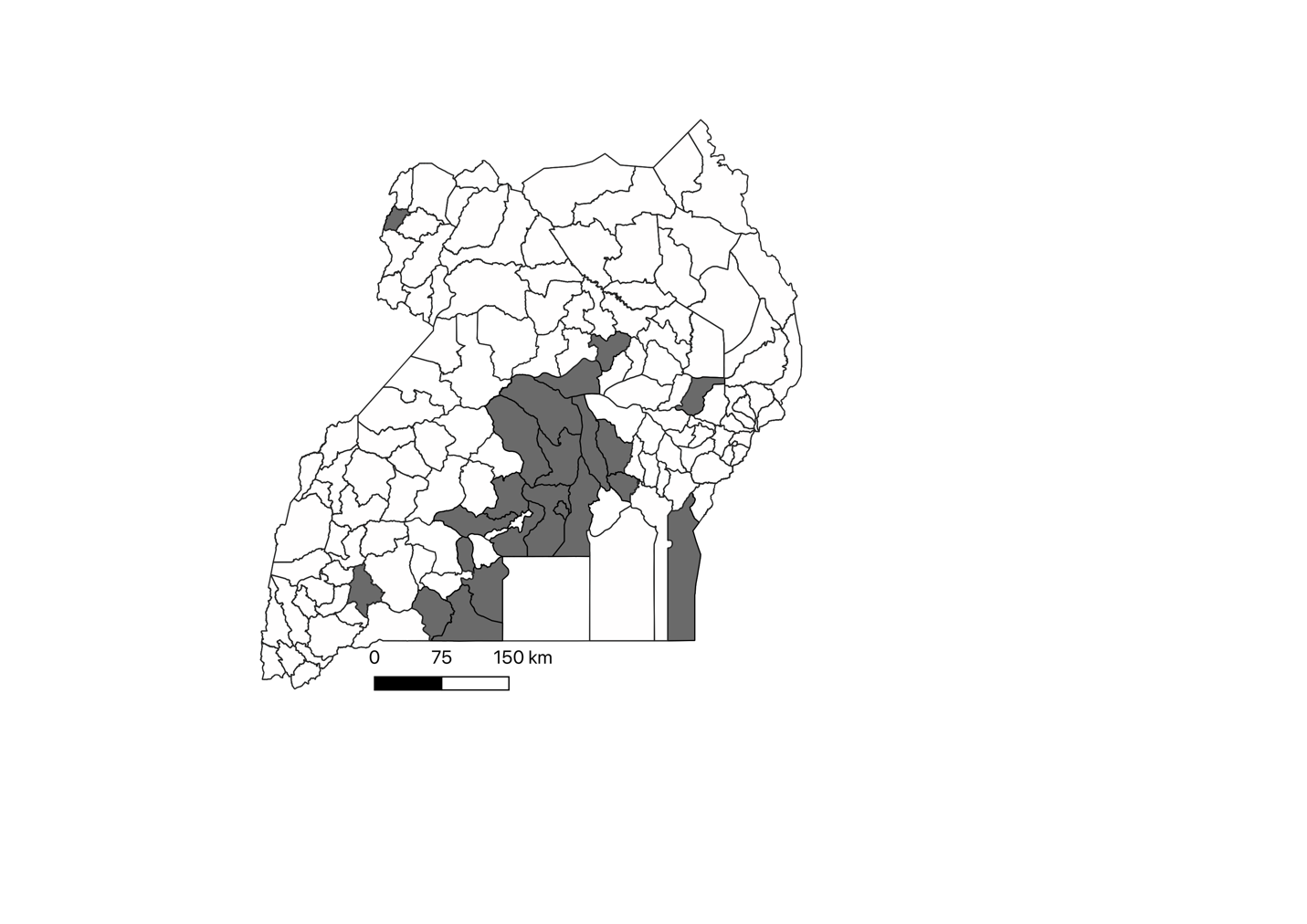


Figure S5: Distribution of Ugandan districts from where pigs sampled at the Lusanja abattoir from May 2021 through June 2022 originated.


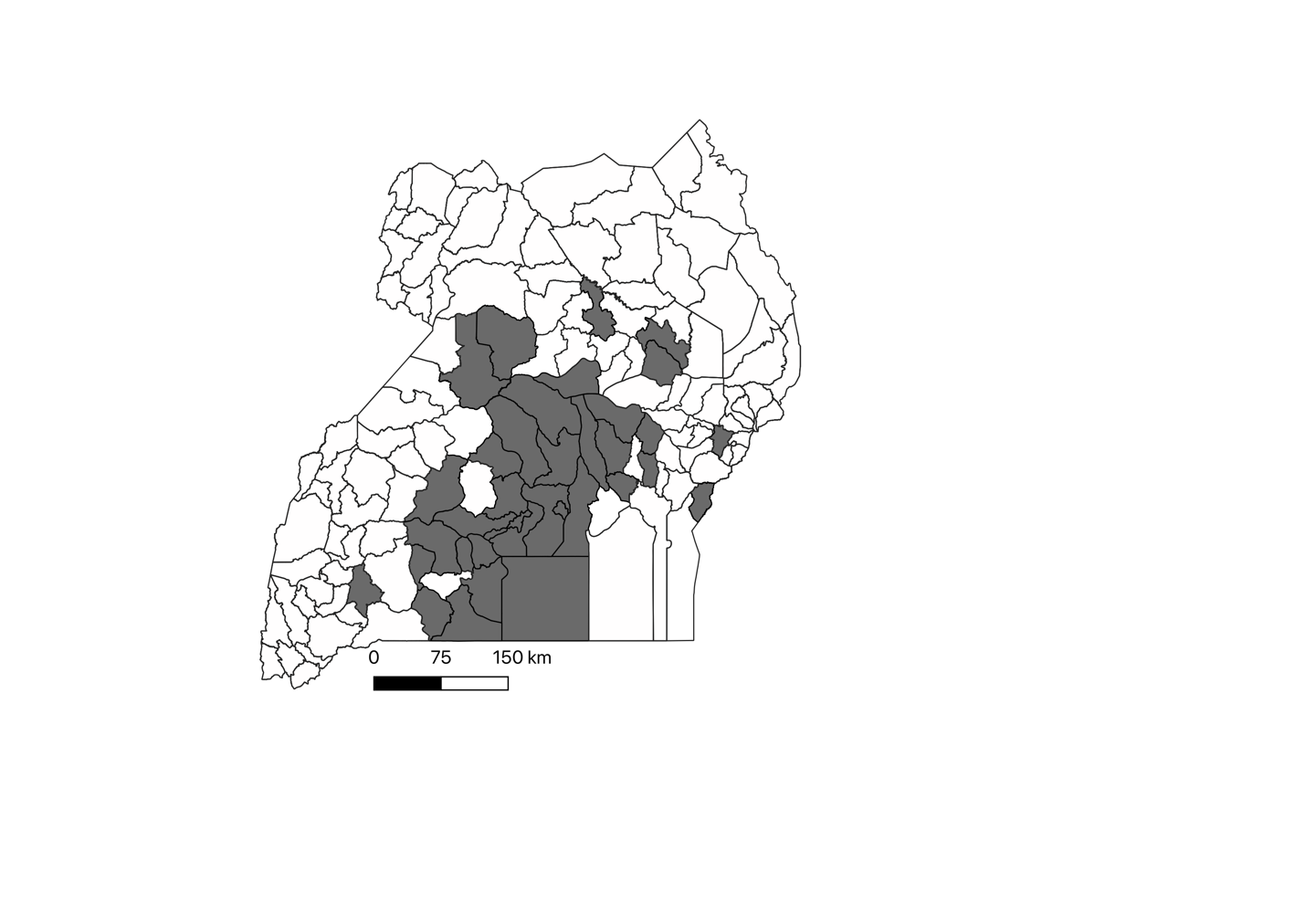


Figure S6: Distribution of Ugandan districts from where pigs sampled at the Wambizi abattoir from May 2021 through June 2022 originated.

Table S2: Distribution of samples and qPCR positivity rates by administrative district from pigs sampled at abattoirs in the greater Kampala metropolitan area from May 2021 through June 2022.

| **District** | **% positivity - Blood** | **% positivity - Spleen** | **% positivity - Lymph nodes** | **% positivity - Tonsil** | **% positivity - Pig** | **# of samples per district** | **% of overall samples** |
| --- | --- | --- | --- | --- | --- | --- | --- |
| Amolatar | 3.2% | 32.1% | 31.3% | 40.6% | 66.7% | 33 | 2.5% |
| Amuria | 100.0% | 0.0% | 100.0% | 100.0% | 100.0% | 1 | 0.1% |
| Apac | 0.0% | 0.0% | 33.3% | 0.0% | 33.3% | 3 | 0.2% |
| Buikwe | 0.0% | 16.7% | 50.0% | 33.3% | 83.3% | 6 | 0.5% |
| Bukomansimbi | 12.5% | 37.5% | 33.3% | 26.7% | 50.0% | 16 | 1.2% |
| Busia | 0.0% | 0.0% | 0.0% | 50.0% | 50.0% | 2 | 0.2% |
| Butambala | 0.0% | -- | 0.0% | 100.0% | 100.0% | 1 | 0.1% |
| Buyende | 50.0% | 80.0% | 40.0% | 80.0% | 100.0% | 6 | 0.5% |
| Dokolo | 0.0% | 33.3% | 33.3% | 0.0% | 50.0% | 6 | 0.5% |
| Gomba | 11.1% | 38.9% | 25.0% | 50.0% | 65.0% | 20 | 1.5% |
| Hoima | 25.0% | 25.0% | 50.0% | 25.0% | 50.0% | 4 | 0.3% |
| Iganga | 0.0% | 45.5% | 54.5% | 54.5% | 81.8% | 11 | 0.8% |
| Jinja | 27.3% | 50.0% | 10.0% | 60.0% | 90.9% | 11 | 0.8% |
| Kalangala | 80.0% | 40.0% | 60.0% | 100.0% | 100.0% | 5 | 0.4% |
| Kaliro | 33.3% | 33.3% | 66.7% | 77.8% | 88.9% | 9 | 0.7% |
| Kalungu | 0.0% | 12.5% | 57.1% | 83.3% | 87.5% | 8 | 0.6% |
| Kampala | 40.0% | 53.8% | 60.0% | 61.5% | 86.7% | 15 | 1.1% |
| Kamuli | 12.5% | 24.4% | 30.2% | 39.5% | 60.4% | 48 | 3.6% |
| Kasanda | 0.0% | 0.0% | 0.0% | 0.0% | 0.0% | 2 | 0.2% |
| Kayunga | 8.6% | 35.7% | 41.5% | 29.8% | 57.6% | 59 | 4.4% |
| Kiboga | 11.5% | 24.0% | 25.0% | 40.0% | 50.0% | 26 | 2.0% |
| Kiryandongo | 33.3% | 33.3% | 33.3% | 33.3% | 100.0% | 3 | 0.2% |
| Kumi | 0.0% | 0.0% | 0.0% | 0.0% | 0.0% | 7 | 0.5% |
| Kyankwanzi | 12.5% | 57.1% | 62.5% | 37.5% | 75.0% | 8 | 0.6% |
| Kyotera | 12.0% | 16.7% | 41.7% | 39.1% | 60.0% | 25 | 1.9% |
| Lira | 0.0% | 50.0% | 0.0% | 50.0% | 33.3% | 6 | 0.5% |
| Luwero | 9.4% | 30.1% | 37.0% | 26.0% | 57.9% | 107 | 8.0% |
| Lyantonde | 0.0% | 0.0% | 66.7% | 60.0% | 60.0% | 5 | 0.4% |
| Maracha | 0.0% | 100.0% | 100.0% | 100.0% | 100.0% | 1 | 0.1% |
| Masaka | 14.2% | 27.6% | 37.1% | 36.2% | 56.5% | 184 | 13.8% |
| Masindi | 22.2% | 28.6% | 12.5% | 33.3% | 44.4% | 9 | 0.7% |
| Mbale | 0.0% | 0.0% | 0.0% | 33.3% | 33.3% | 3 | 0.2% |
| Mbarara | 50.0% | 50.0% | 50.0% | 50.0% | 50.0% | 2 | 0.2% |
| Mityana | 25.0% | 33.3% | 9.1% | 33.3% | 33.3% | 12 | 0.9% |
| Mpigi | 22.6% | 36.2% | 43.9% | 39.7% | 64.5% | 62 | 4.7% |
| Mubende | 35.7% | 50.0% | 50.0% | 64.3% | 85.7% | 14 | 1.1% |
| Nakasongola | 41.2% | 41.2% | 82.4% | 81.3% | 94.1% | 17 | 1.3% |
| Namayingo | 0.0% | 0.0% | 0.0% | 0.0% | 0.0% | 1 | 0.1% |
| Rakai | 22.2% | 33.3% | 87.5% | 62.5% | 77.8% | 9 | 0.7% |
| **District** | **% positivity - Blood** | **% positivity - Spleen** | **% positivity - Lymph nodes** | **% positivity - Tonsil** | **% positivity - Pig** | **# of samples per district** | **% of overall samples** |
| Wakiso | 18.1% | 36.2% | 37.1% | 39.0% | 61.0% | 359 | 26.9% |
| Unknown | 14.3% | 26.8% | 38.9% | 29.6% | 50.0% | 58 | 4.4% |
